# A device-independent quantum key distribution system for distant users

**DOI:** 10.1038/s41586-022-04891-y

**Published:** 2022-07-27

**Authors:** Wei Zhang, Tim van Leent, Kai Redeker, Robert Garthoff, René Schwonnek, Florian Fertig, Sebastian Eppelt, Wenjamin Rosenfeld, Valerio Scarani, Charles C.-W. Lim, Harald Weinfurter

**Affiliations:** 1 Fakultät für Physik, Ludwig-Maximilians-Universität, München, Germany; 2 Munich Center for Quantum Science and Technology (MCQST), München, Germany; 3 Naturwissenschaftlich-Technische Fakultät, Universität Siegen, Siegen, Germany; 4 Department of Electrical & Computer Engineering, National University of Singapore, Singapore, Singapore; 5 Centre for Quantum Technologies, National University of Singapore, Singapore, Singapore; 6 Department of Physics, National University of Singapore, Singapore, Singapore; 7 Max-Planck Institut für Quantenoptik, Garching, Germany; 8 Present Address: JPMorgan Chase, Singapore, Singapore

**Keywords:** Quantum information, Atomic and molecular interactions with photons

## Abstract

Device-independent quantum key distribution (DIQKD) enables the generation of secret keys over an untrusted channel using uncharacterized and potentially untrusted devices^1–9^. The proper and secure functioning of the devices can be certified by a statistical test using a Bell inequality^10–12^. This test originates from the foundations of quantum physics and also ensures robustness against implementation loopholes^13^, thereby leaving only the integrity of the users’ locations to be guaranteed by other means. The realization of DIQKD, however, is extremely challenging—mainly because it is difficult to establish high-quality entangled states between two remote locations with high detection efficiency. Here we present an experimental system that enables for DIQKD between two distant users. The experiment is based on the generation and analysis of event-ready entanglement between two independently trapped single rubidium atoms located in buildings 400 metre apart^14^. By achieving an entanglement fidelity of ${\mathcal F} \,\ge 0.892(23)$ and implementing a DIQKD protocol with random key basis^15^, we observe a significant violation of a Bell inequality of *S* = 2.578(75)—above the classical limit of 2—and a quantum bit error rate of only 0.078(9). For the protocol, this results in a secret key rate of 0.07 bits per entanglement generation event in the asymptotic limit, and thus demonstrates the system’s capability to generate secret keys. Our results of secure key exchange with potentially untrusted devices pave the way to the ultimate form of quantum secure communications in future quantum networks.

## Abstract (web summary)

A system based on trapped rubidium atoms for generating quantum secure keys between distant users is presented, which could operate in a device-independent fashion.

## Main

Secure communication over public channels requires the users to share a common secret key. Today, this crucial task faces major challenges from quantum-based attacks and implementation vulnerabilities. A promising solution is to use quantum key distribution (QKD), which uses the laws of quantum physics to assess eavesdropping attempts on the public channel^16,17^. However, in its standard form, QKD is prone to implementation side channels, like all modern information systems^13,18^. In particular, the security of QKD is also based on the mathematical models of the devices, so it is absolutely essential that the quantum devices are behaving as specified during the protocol execution.

Device-independent QKD^1–9^ (DIQKD) is an advanced form of QKD. First proposed by Mayers and Yao^1^, it warrants the proper and secure functioning of the underlying devices by a Bell test^11^, in which the users only need to analyse their input–output measurement data to establish an upper limit on the amount of information that an eavesdropper could have gained during the protocol. Importantly, this verification step eliminates the need to characterize the quantum devices and hence DIQKD is naturally robust against implementation flaws.

To implement DIQKD, a system is required that distributes high-quality entangled states with high detection efficiency between two remote locations. More specifically, the system needs to achieve both high Bell violation and low quantum bit error rate (QBER) to generate secret keys. State-of-the-art systems can achieve high Bell violations between distant particles^14,19–21^, but are not good enough to generate a secret key in the device-independent setting^22^. In a recent effort to relax the system requirements various improved designs of the original DIQKD protocol^2,3^ were introduced, for example, on the basis of noisy preprocessing^23^, randomized key settings^15^ and random post-selection^24^. Simultaneously to this work, two proof-of-concept DIQKD experiments were performed: one demonstrated finite-key distribution over 2 m using trapped ions^25^ and the other verified that a photonic implementation over up to 220 m of fibre is within reach^26^.

Here, we report on an experimental system that enables DIQKD between two distant users. It combines experimental advances in a previous loophole-free Bell test experiment^14^ with the DIQKD protocol proposed in ref. ^15^. The quantum channel is formed by two single ^87^Rb atoms, trapped and manipulated individually in buildings approximately 400 m line-of-sight apart. More specifically, entanglement between the two atoms is created through an event-ready entanglement swapping scheme, which is performed across a 700 m long optical fibre connecting the two buildings. Substantial improvements in the entanglement quality, entanglement generation rate and noise tolerance of the protocol enable the system to achieve a positive secret key rate (the ratio of achievable secret key length to the total number of heralded events) of 0.07 bits in a fully device-independent configuration.

## DIQKD protocol

**Fig. 1:**
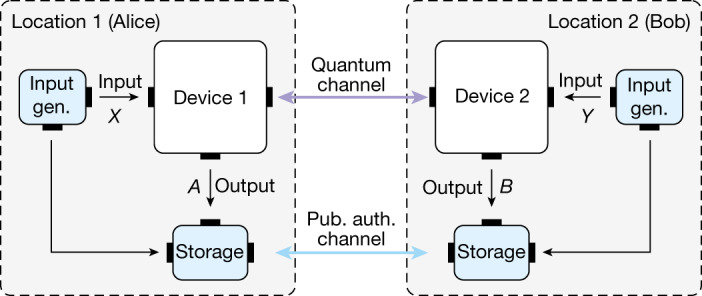
Schematic of a DIQKD scheme. Each of the two parties, Alice and Bob, holds QKD devices, which are connected by a quantum channel. The devices receive the inputs *X* and *Y*, and respond with outputs *A* and *B*, respectively. To run the protocol each party needs a trusted supply of inputs and a trusted local storage unit to store both output and inputs. Additionally, a trusted authenticated public channel (pub. auth. channel) between the two parties is necessary for exchange of information during post-processing. gen., generation.

Let us first review the basic assumptions of DIQKD. The two users, Alice and Bob, should (1) each hold a device that is able to receive an input and then respond with an unambiguous output that can be used to generate a secure key (Fig. 1). The communication between their devices is limited to what is necessary to generate a secure key, namely, (2) the users control when their respective devices communicate with each other^27^; and (3) the devices do not send unauthorized classical information to an eavesdropper. Finally, as it is with any QKD protocol, it is required that (4-a) quantum mechanics is correct, (4-b) the users’ inputs are private and random and (4-c) the users are connected by an authenticated classical channel and use trusted post-processing methods. For more details, we refer the interested reader to Supplementary Appendix A.

The DIQKD protocol considered here is similar to the original DIQKD protocol^2,3^, except that two measurement settings are used for key generation instead of one. Importantly, in doing so, the protocol can tolerate more system noise—the critical QBER increases from 0.071 to 0.082 (ref. ^15^). The protocol considers that Alice and Bob each hold a device, which are connected by a quantum channel (Fig. 1). In each *i*th of *N* measurement rounds, one of four different inputs ${X}_{i}\in \{0,1,2,3\}$ is given to Alice’s device, whereas Bob’s device receives one of two possible values ${Y}_{i}\in \{0,1\}$. The input for each round is provided by a trusted local source of randomness. Both devices output two possible values, ${A}_{i}\in \{\uparrow ,\downarrow \}$ at Alice’s side and ${B}_{i}\in \{\uparrow ,\downarrow \}$ at Bob’s side. The input and output values are recorded and stored in independent, local secured storage.

After *N* rounds classical post-processing starts, with Alice and Bob revealing their inputs for each round over an authenticated public channel. For the rounds with differing input settings, that is, ${X}_{i}\in \{2,3\}$ together with ${Y}_{i}\in \{0,1\}$, the outputs are shared over the public channel to compute the Clauser–Horne–Shimony–Holt (CHSH)^28^ value using

1 $$S:= {E}_{2,1}\,-\,{E}_{2,0}\,-\,{E}_{3,0}\,-\,{E}_{3,1},$$

where the correlation functions are defined as ${E}_{X,Y}:= {p}_{X,\,Y}^{A=B}-{p}_{X,\,Y}^{A\ne B}$. Probabilities of the form ${p}_{X,\,Y}^{A,B}$ are estimated by the ratio ${N}_{X,\,Y}^{A,B}/{N}_{X,Y}$ of the number of rounds with outcomes (*A*, *B*) for input combination (*X*, *Y*), to the total number of rounds with those inputs. Provided that the devices share a sufficiently entangled state, the Bell inequality can be violated, that is, *S* > 2.

The raw data are sifted so that only the outputs of measurement rounds with identical input settings are kept for further processing. The QBERs for both key settings are denoted by ${Q}_{0}={N}_{0,0}^{A\,=\,B}/{N}_{0,0}$ for *X*_*i*_ = *Y*_*i*_ = 0 and ${Q}_{1}={N}_{1,1}^{A\,=\,B}/{N}_{1,1}$ for *X*_*i*_ = *Y*_*i*_ = 1. Note that the key pairs are anticorrelated when using anticorrelated entangled states. Both the QBERs (*Q*_0_, *Q*_1_) and the CHSH value *S* are used to determine the amount of information about the sifted key that could have been obtained by an eavesdropper^29^. Next, by applying a technique known as leftover hashing, the eavesdroppers (quantum) information about the final key can be reduced to an arbitrary low level, defined by the security error of the protocol^30^. In this experiment, we focus on estimating the asymptotic security performance of the considered DIQKD protocol. For this purpose, we note that in the asymptotic limit and in case of a depolarizing quantum channel, positive key rates can be achieved when the expected CHSH value satisfies *S* > 2.362 (or equivalently, *Q* < 0.082 with *Q*_0_ = *Q*_1_ = *Q*)^15^.

## Quantum network link

**Fig. 2:**
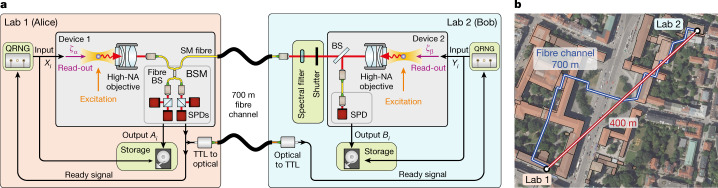
Overview of the DIQKD system. **a**, Alice’s equipment (Device 1 in Lab 1) is formed by a single-atom trap and a BSM set-up. Bob (Device 2 in Lab 2) uses a second single-atom trap together with a 90:10 (T:R) beam splitter (BS) and a single-photon detector (SPD). Each trap set-up contains a high numerical aperture (NA) objective to optically trap a single atom and collect atomic fluorescence into a single-mode (SM) fibre. The atoms are entangled in an event-ready scheme by synchronously exciting them, after which the spontaneously emitted photons are collected by high-NA objectives and guided to the BSM. Here, a coincidental photon detection on two detectors in the same output arm of the fibre BS heralds the entangled atom–atom state $|{\Psi }^{+}\rangle$, which is announced to both users by a ‘ready’ signal. After receiving the ready signal, two quantum random number generators (QRNGs) select the inputs to the devices, determining the polarization of a read-out pulse in a state-selective ionization scheme. The binary output of the devices is determined from a fluorescence measurement of atom presence after the ionization attempt, as ionized atoms are lost from the trap. The inputs and outputs of each round are stored locally using a trusted storage. In Lab 2 a spectral filter and shutter are implemented to avoid leakage of the inputs and outputs of the device. **b**, Map showing the main campus of the LMU in Munich, indicating the locations of the two laboratories. Map data in **b** are from Bayerische Vermessungsverwaltung .

A quantum network link (QNL) generates the entanglement to implement the DIQKD protocol. In our set-up, event-ready entanglement is generated between two optically trapped single ^87^Rb atoms located in laboratories 400 m apart and connected by a 700 metre long optical fibre channel (Fig. 2). The atoms act as quantum memories in which a qubit is encoded in the Zeeman substates of the $5{{\rm{S}}}_{1/2}|F=1,{m}_{F}=\pm 1\rangle$ ground state, with *m*_*F*_ = +1 and *m*_*F*_ = −1 designated as computational basis states, ${|\uparrow \rangle }_{z}$ and ${|\downarrow \rangle }_{z}$, respectively, and where the quantization axis $\hat{z}$ is defined by the fluorescence collection set-up.

**Fig. 3:**
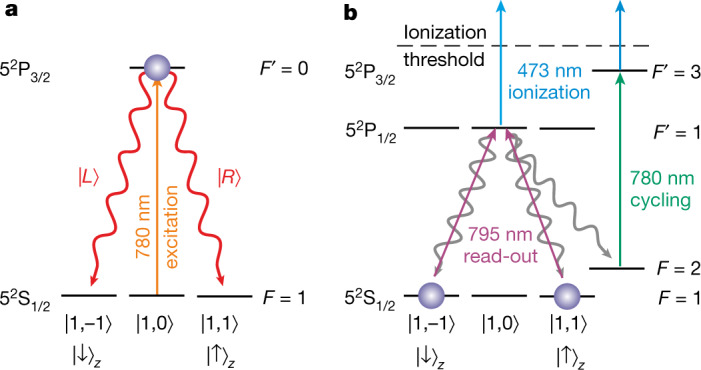
Schematics of the entanglement generation and atomic-state read-out schemes. **a**, An entangled atom–photon state is generated by the spontaneous emission of a photon subsequent to excitation of the atom. Decay from the state ${5}^{2}{{\rm{P}}}_{3/2}|F{\prime} =0,{m}_{{F}^{{\prime} }}=0\rangle$ results in an entangled atom–photon state ${| \Psi \rangle }_{AP}=1/\sqrt{2}({| \downarrow \rangle }_{x}| H\rangle +{| \uparrow \rangle }_{x}| V\rangle )$^41^, where ${|\uparrow \rangle }_{x}:= 1/\sqrt{2}({|\uparrow \rangle }_{z}+{|\downarrow \rangle }_{z})$ (respectively ${|\downarrow \rangle }_{x}:= i/\sqrt{2}({|\uparrow \rangle }_{z}-{|\downarrow \rangle }_{z})$) and $|H\rangle$ and $|V\rangle$ denote parallel and orthogonal linear polarizations with respect to the optical table, respectively, with $|V\rangle := 1/\sqrt{2}(|L\rangle +|R\rangle )$ and $|H\rangle := i/\sqrt{2}(|L\rangle -|R\rangle )$. **b**, The atomic qubit state is read out by a state-dependent ionization scheme. First, a certain superposition of the qubit state is excited to the 5^2^P_1/2_ level depending on a respective polarization of the so-called read-out laser light (*λ* = 795 nm). The excited atom is ionized by a bright second laser applied simultaneously at *λ* = 473 nm. If the atom decays to the state ${5}^{2}{{\rm{S}}}_{1/2}|F=2\rangle$ before it is ionized, it is excited to the state ${5}^{2}{{\rm{P}}}_{3/2}|F{\prime} =3\rangle$ with the third excitation laser at *λ* = 780 nm, which is ionized as well.

The two distant atoms are entangled using an entanglement swapping protocol^31^. The sequence starts by synchronously exciting the single atom in each trap to the state ${5}^{2}{{\rm{P}}}_{3/2}|F{\prime} =0,{m}_{{F}^{{\prime} }}=0\rangle$; when decaying to the ground state, each of the atomic qubits becomes entangled with the polarization of the respective spontaneously emitted single photon (Fig. 3a). The two photons are then guided to a Bell-state measurement (BSM) set-up using two-photon interference. Projection of the photons onto a $|{\Psi }^{+}\rangle$ state heralds the creation of the maximally entangled atom–atom state

2 $${|{\Psi }^{+}\rangle }_{AB}=\frac{{|\uparrow \rangle }_{x,A}{|\downarrow \rangle }_{x,B}+{|\downarrow \rangle }_{x,A}{|\uparrow \rangle }_{x,B}}{\sqrt{2}}.$$

Given a successful projection, a ‘ready’ signal is sent to the trap set-ups, initiating the next measurement round for which, depending on input values *X*_*i*_ and *Y*_*i*_, the two atomic qubits are independently analysed by state-selective ionization (Fig. 3b)^32^. There, a particular state of the atomic qubit is ionized and leaves the trap depending on the polarization $\zeta =\,\cos (\gamma )V+{{\rm{e}}}^{-i\varphi }\,\sin (\gamma )H$ of a read-out laser pulse (*γ* = *α* for Alice’s and *γ* = *β* for Bob’s device). If the atom is still in the trap, it is thus projected onto the state

3 $$| D\rangle =\,\sin (\gamma ){| \downarrow \rangle }_{x}-{{\rm{e}}}^{-i\varphi }\,\cos (\gamma ){| \uparrow \rangle }_{x}\,=\,| \uparrow \rangle .$$

The presence of the atom is then tested using fluorescence collection at 780 nm, which yields the final measurement outcomes *A*_*i*_ and *B*_*i*_, respectively. On Alice’s side, the single-photon detectors of the BSM detect the fluorescence of the atom, whereas on Bob’s side an unbalanced beam splitter directs a small fraction of the florescence light onto a single single-photon detector (Fig. 2). As the results are reported every time, the detection efficiencies of Alice’s and Bob’s measurements are effectively one. Any component loss or ionization inefficiency contributes to the noise in the quantum channel.

The requirements for DIQKD implementation are less stringent with the newly proposed protocols; however, substantial improvements over existing loophole-free Bell experiments were still required. To that end, we enhanced the entanglement generation rate, coherence of atomic states and entanglement swapping fidelity (Methods).

## DIQKD implementation

The independent random inputs to the devices (requirement (4-b)) are provided by independent quantum random number generators with a bias lower than 10^−5^ located in each laboratory^14,33^. At Alice’s side, two random bits are used to select the input, whereas at Bob’s side only one random bit is used, leading to uniformly distributed input combination choices. For the generated entangled state equation (2) and the atomic-state measurement scheme equation (3), the input values $X\in \{0,1,2,3\}$ convert to measurement angles $\alpha \in \{-{22.5}^{\circ },+\,{22.5}^{\circ },-\,{45}^{\circ },{0}^{\circ }\}$ for Alice’s device, whereas $Y\in \{0,1\}$ translates to $\beta \in \{+{22.5}^{\circ },-\,{22.5}^{\circ }\}$ for Bob’s device. The capability for fast switching between various read-out settings is achieved by overlapping multiple read-out beams with different polarization and individually controllable intensities^14^. The outputs $A,B\in \{\uparrow ,\downarrow \}$ are derived from the fluorescence counts after the state-selective ionization. Finally, the users’ inputs and outcomes are stored in two independent, trusted secure storages (requirement 4-c).

Unauthorized incoming and outgoing communication of the laboratories can be prevented with prudent steps (requirements (2) and (3)). Especially on Bob’s side, extra measures are taken to prevent information leakage from the laboratory: a free-space shutter is closed during the read-out process to keep the leakage of fluorescence light into the optical fibre and the outside environment to well below one photon per read-out event (Fig. 2), and the trap is always emptied before reopening the shutter. Owing to the approximate 5 ms reaction time of the shutter, a spectral filter (10^−6^ transmission at 795 nm) is deployed to block the read-out pulse after interacting with the atom and to prevent unintentional transmission of the read-out setting. For Alice’s side, such countermeasures are not needed as the BSM set-up already serves as a natural blocker^34^.

## System measurements and performance

**Fig. 4:**
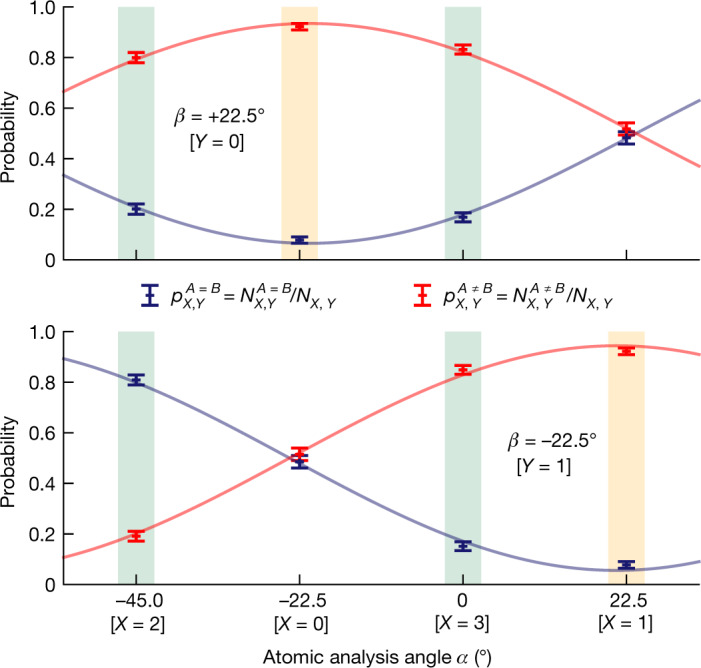
Atom–atom state correlations. The correlations *E*_*X,Y*_ are obtained from the correlation (blue) and anticorrelation (red) probabilities of the device outputs for the eight input combinations. The data are fitted with sinusoidal functions estimating visibilities of 0.869(25) and 0.888(45). The settings for *X* = 2 or *X* = 3 (green background) contribute to the evaluation of the Bell parameter *S* = 2.578(75), whereas the QBER *Q* = 0.078(9) follows from settings with *X* = *Y* (yellow background). The error bars indicate statistical errors of one standard deviation. *N* = 3,342.

The inputs and outputs of the devices were recorded for *N* = 3,342 rounds over a measurement period of 75 h. The resulting output (anti)correlation probabilities for the eight different input combinations, that is, ${N}_{X,\,Y}^{A\,=\,B}/{N}_{X,Y}$ and ${N}_{X,\,Y}^{A\,\ne \,B}/{N}_{X,Y}$, are shown in Fig. 4.

It is instructive to first review the increased performance of the QNL independently of the DIQKD protocol. Here, the figure of merit is the fidelity of the observed entangled atom–atom state relative to a maximally entangled state. By fitting the data (Fig. 4) with sinusoidal functions, the estimated visibility for input combinations *X* = 2, 0, 3, 1 and *Y* = 0 (respectively *X* = 2, 0, 3, 1 and *Y* = 1) is 0.869(25) (respectively 0.888(45)). Then, averaging the found visibilities and taking into account that a third atomic ground-level spin state can be populated $({5}^{2}{{\rm{S}}}_{1/2}|F=1,{m}_{F}=0\rangle )$, a lower bound on the fidelity is given by ${\mathcal F} \ge 0.892(23)$ (ref. ^35^).

The CHSH value is found to be *S* = 2.578(75) using equation (1) with *E*_2,0_ = −0.599(41), *E*_3,0_ = −0.664(36), *E*_2,1_ = 0.618(39) and *E*_3,1_ = −0.697(35). The QBERs are given by the correlation data for *X* = *Y*, that is, *Q*_0_ = 0.0781(127) and *Q*_1_ = 0.0777(132), which gives an average error rate of *Q* = 0.078(9). For the considered DIQKD protocol and the uniformly distributed measurement settings, the observed *S* value and QBER result in a secret key rate of 0.07 bits in the asymptotic limit, out of a maximum achievable value of 0.25—showing that the system is capable of performing DIQKD between two users 400 m apart. To quantify the confidence of this estimate, we assume that underlying input–output probability distributions are independent and identically distributed and use standard Bayesian methods to determine the uncertainties of the estimated parameters. We find that taking the worst-case estimates of *S* (2.4256), *Q*_1_ (0.107) and *Q*_2_ (0.107) using a common probability error of 3% still give a positive rate. We note that, thanks to the high-quality entanglement, also the original DIQKD protocol^2,3^ achieves a positive key rate for the observed *S* and *Q*_0_ (or *Q*_1_), but only for a larger common probability error.

**Fig. 5:**
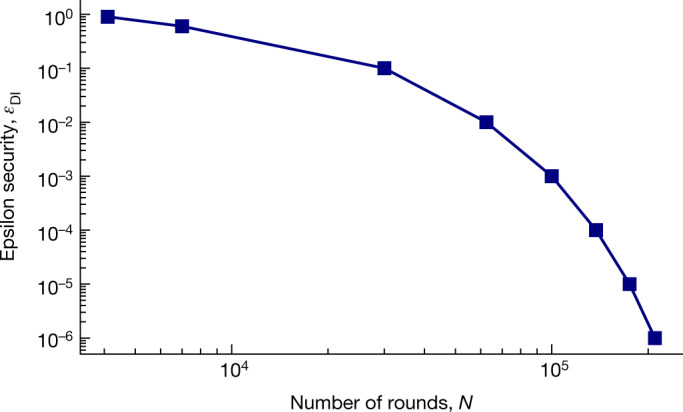
Finite-key simulation for the robust DIQKD protocol. Shown is the minimum number of rounds, that is, block length, required to distribute a finite key with a certain epsilon security, considering collective attacks and uniformly distributed measurement settings. The channel parameters *S*, *Q*_0_ and *Q*_1_ are set to the observed values in the experiment. A non-asymptotic security of *ε*_DI_ = 10^−5^ is considered to be realistic for cryptography applications.

In addition, using state-of-the-art finite-key analysis^30^ for the protocol, we find that for a typical security error value of *ε*_DI_ = 10^−5^ a secure key can be obtained with a minimum block length of 1.75 × 10^5^, as shown in Fig. 5. Here, *ε*_DI_ is the security error of the protocol and can be seen as the probability that the protocol fails in its task, for example, that the final key pair is not secret^36^. In the simulation, we consider collective attacks, an error correction efficiency of 1.15 and uniformly distributed measurement settings for Alice and Bob.

## Discussion and outlook

In this work, we present an experimental system that is capable of achieving positive asymptotic key rates between users separated by 400 m line-of-sight (700 m fibre length) in a fully device-independent setting. Although the current set-up outperforms existing loophole-free Bell set-ups, there are still several areas that require improvements for implementing DIQKD with finite-key security and longer reach.

For one, a higher event rate is required to obtain finite-key security within a practical time. The event rate critically depends on the entanglement generation efficiency and the repetition rate. To increase the former, several improvements are possible, for example, improving the BSM set-up fidelity to include the $|{\Psi }^{-}\rangle$ state projection would increase the entanglement generation rate by a factor of 2. Furthermore, it is possible to scale up the number of atom traps using multidimensional arrays^37–39^, which, combined with time multiplexing techniques^40^, could increase the event rate by several orders of magnitude (Supplementary Appendix H).

Another direction is to improve the reach of the QNL. Here, a limiting factor is attenuation loss of the 780 nm photons in long optical fibres, which is already 50% for a 700 m long link. To overcome losses in longer fibre links, a promising solution is to convert the entangled single photons to the low-loss telecom band by polarization-preserving quantum frequency conversion^32^. Recent results demonstrate extension of the QNL to 33 km fibre length^35^ and show that high-quality entanglement over distances up to 100 km is achievable.

In summary, our results represent a major step towards the goal of ultimate secure communication based solely on the laws of physics. They indicate that state-of-the-art quantum links are capable of generating secret keys. Moreover, they show that future quantum networks distributing entanglement between their nodes can harness this quantum advantage, making DIQKD the standard for secure communications.

## Methods

### Increased entanglement generation rate

Custom-designed high-numerical aperture objectives are installed in each trap to increase the single-photon collection efficiency by a factor greater than 2.5. This ultimately leads to an atom–atom entanglement generation efficiency of 0.49 × 10^−6^ following an excitation attempt. Together with a duty cycle of approximately ½ and a repetition rate of the entanglement generation tries of 52 kHz, this results in an event rate of 1/82 s^−1^. Note that for event-ready entanglement generation schemes the repetition rate of the experiment is limited by the communication times between the two devices and the BSM^35^. For DIQKD protocols, this results in a trade-off between the maximum separation of the users and the achieved secret key rate.

### Atomic coherence time

The coherence and stability of the atomic qubit states are limited by the fluctuations of local magnetic fields and position-dependent vector light shifts, which are introduced by the tight focus of the optical dipole traps. The latter is especially crucial as it enables a high-fidelity state measurement only when the atom has completed a full transverse oscillation in the trap^42^. Here, the better optical components of the new collection set-up, which is also used to focus the trapping laser, improve the spatial symmetry of the trapping potential and thereby enable a better cancellation of dephasing effects. In combination with lowering the atom temperatures and applying a magnetic bias field, this extends the coherence time to approximately 330 μs. This results in a lower bound on the atom–photon entanglement fidelity of 0.952(7) and 0.941(7) (relative to a maximally entangled state) for Alice’s and Bob’s set-ups, respectively. We refer the interested reader to Supplementary Appendix B for more details.

### BSM fidelity

The quality of the entangled atom–atom state is further improved by optimizing the two-photon interference of the BSM on the basis of a rigorous analysis of the atom–photon entanglement generation process. Here, the multilevel structure of ^87^Rb, the finite duration of the excitation pulse and experimental imperfections lead to the possibility of two-photon emission from one atom. Crucially, these multiphoton events reduce the fidelity of the BSM result. To overcome this, only photons that are emitted after the end of the previous excitation pulse are accepted in the BSM. This time filtering reduces the entanglement generation rate by a factor of 4 (resulting in the entanglement generation rate mentioned before), but greatly increases the fidelity of the generated state (see Supplementary Appendix C for more details).

## Online content

Any methods, additional references, Nature Research reporting summaries, source data, extended data, supplementary information, acknowledgements, peer review information; details of author contributions and competing interests; and statements of data and code availability are available at 10.1038/s41586-022-04891-y.

## Supplementary information

Supplementary Information Supplementary figures, Appendices A–H, tables and references.

## Data Availability

The datasets generated and/or analysed during the experiment are available from the corresponding authors on reasonable request.

## Code availability

The code supporting the plots within this paper is available from the corresponding authors upon reasonable request.
