# Supplementary material for: A device-independent quantum key distribution system for distant users
Source: Nature. 2022 Jul 27;607(7920):687–91. doi: 10.1038/s41586-022-04891-y (PMC9329124; doi:10.1038/s41586-022-04891-y)
Supplement: Supplementary file 1 — Supplementary figures, Appendices A–H, tables and references. [file 41586_2022_4891_MOESM1_ESM.pdf]

nature portfolio

https://doi.org/10.1038/s41586-022-04891-y

Supplementary information

A device-independent quantum key distribution system for distant users

In the format provided by the authors and unedited

Supplementary Information for: "A device-independent quantum key distribution system for distant users"

Wei Zhang,[1,2,*] Tim van Leent,[1,2,*] Kai Redeker,[1,2,*] Robert Garthoff,[1,2,*]
René Schwonnek,[3,4] Florian Fertig,[1,2] Sebastian Eppelt,[1,2] Wenjamin Rosenfeld,[1,2]
Valerio Scarani,[5,6] Charles C.-W. Lim,[3,5,†] and Harald Weinfurter[1,2,7,‡]

[1]*Fakultät für Physik, Ludwig-Maximilians-Universität, München, Germany*
[2]*Munich Center for Quantum Science and Technology (MCQST), München, Germany*
[3]*Department of Electrical & Computer Engineering, National University of Singapore, Singapore*
[4]*Naturwissenschaftlich-Technische Fakultät, Universität Siegen, Germany*
[5]*Centre for Quantum Technologies, National University of Singapore, Singapore*
[6]*Department of Physics, National University of Singapore, Singapore*
[7]*Max-Planck Institut für Quantenoptik, Garching, Germany*

Appendix A: In which sense DIQKD is "device-independent": assumptions and requirements

The name *device-independent* QKD suggests that secrecy can be guaranteed "without any knowledge of the device". Such a compact statement may lead (and has actually led) to misinterpretations. It requires qualification, which we split in four requirements already mentioned in the main text. The qualified claim of DIQKD is: given devices whose inputs, outputs and interfaces are controlled by the users [requirements (1) and (2)], secrecy is guaranteed under the obvious assumption that the secret does not leak out of the secure locations (3), as well as under the requirements needed for any QKD protocol (4). In this Appendix we elaborate on these matters.

a. Scenario – Two parties, named Alice and Bob, want to establish a secret key in order to exchange secret messages. A *secret key* is a list of bits that is identical between Alice and Bob, and guaranteed to be known only to them—in other words, it is *shared secret randomness*. The adversary, who may be actually trying to break the protocol, is called Eve for narrative convenience. Quantum key distribution (QKD) is a practical solution for this task. The resources required in QKD are:

- Secure locations: it must be assumed that the two environments in which Alice and Bob operate are not compromised. However, practically speaking, this can never be guaranteed unconditionally; the level of paranoia is subjective, for this involves individuals and methods which go beyond what quantum physics can certify. As such, one can only enforce the best possible known methods in practice to prevent unauthorised information leakage. For example, in our experiment, we used a free-space shutter to prevent fluorescence light from leaking out into an optical fibre leaving the laboratory.

- An unlimited (for all practical purposes) amount of local randomness, i.e. the possibility of generating strings of bits that are unknown to anyone else (in this case, even to the other authorised partner). These will constitute the "trusted inputs" to the devices.

- An authenticated public channel for classical communication between them. In order to authenticate the channel, Alice and Bob must possess some shared randomness prior to the start. Thus, QKD is actually quantum key *expansion*: the amount of secret key generated by the protocol should exceed the amount that is consumed to authenticate the channel and for classical post-processing.

- Last but not least, the actual devices that create and process the quantum information, and the quantum channel connecting them. *The "device-independence" of DIQKD means that these devices can be dealt with as black boxes.* Explicitly, the security assessment does not rely on the characterisation and modeling of any of their inner workings and dimensions, not even the type of quantum system and measurements that are actually performed.

Any QKD protocol essentially starts with the distribution and measurement of quantum signals. This part consists of well defined rounds, whereby each round consists of one pair of inputs and outputs for each device. After

* These authors contributed equally
† charles.lim@nus.edu.sg
‡ h.w@lmu.de

accumulating a certain amount of rounds the device inputs are shared over the trusted public channel. Certain input combinations are then used to generate the *sifted (or raw) key*, while others are used to estimate the features of quantum mechanics used to bound Eve's information on the outputs. It is then possible to proceed with error correction and privacy amplification protocols, and extract a *final key* on which Eve has no information. These steps also require adequate and trusted methods that fit to the actual implementation and performance of the DIQKD setup, in order to not distort the information theoretical security.

For DIQKD, the feature of quantum theory used to bound Eve's information is *the violation of a Bell inequality* [1–5]. A Bell inequality test has its own set of requirements, failure to comply with those leads to famous loopholes [6, 7]. First, the requirement of locality, ensuring that the process generating the output in one device is independent of the input and the process of the other. Second, in each round the inputs should be random for the devices. Notice that this is slightly different from the analog requirement of QKD: for QKD, the local input should be random for Eve but might be known by the device; for Bell alone, the local input may be publicly known, as long as it is random for the device. In DIQKD, the local randomness should therefore be *random both for Eve and for the device.* Among other loopholes that may invalidate a Bell test, by far the most important and relevant here is the "detection loophole". To avoid it, one must not assume fair sampling in case of imperfect detection efficiencies: rather, there must be an output of the device for every input (if the detector failed to detect, the output must be generated according to some other local recipe: this will of course reduce the correlations, but won't compromise the soundness of the test). In fact, it is the detection loophole opened by losses that makes it very challenging to implement DIQKD with purely optical setups.

Finally, even though the devices are black boxes, for secrecy one should require that they do not leak any information. For one, the provider of the devices should be trusted as honest: if they are colluding with Eve, surely they have hidden somewhere a small emitter that might leak the key at the end of the protocol (or in later instances). On a more technical level, these devices must be open to the world through the quantum channel (the quantum signals, however uncharacterised, must be able to enter the device). One must then assume that no information leaks out through that port, while open [8]. Note that under these assumptions closing the locality loophole does not increase the security of the protocol [3]. By enforcing that the devices do not leak information, the process generating the output in one device is already independent of the input and the process of the other. Once again, the assumption of no-leakage from the secure location is a requirement for all forms of secrecy. We just brought up possible forms of leakage that are worth mentioning, given the danger of exaggerations associated with the words "device-independence".

Based on what we said, we can summarize the requirements for DIQKD in the following four (order does not indicate importance):

(1) The used system consists of two separated devices, the devices receive an input and respond with a well defined output, and the protocol is split into well defined rounds;

(2) Alice and Bob control when the devices communicate with each other;

(3) The devices do not send classical information to an possible eavesdropper;

(4-a) Quantum theory is correct;

(4-b) Each device is supplied with trusted inputs independent and unknown to an possible attacker (Eve);

(4-c) Alice and Bob are connected via an authenticated channel, employ trusted local storage units, and use appropriate post processing.

b. Experimental requirements– Each of the listed premises has different consequences for an implementation of DIQKD. (4-a) is obvious for any kind QKD. It means that if the world is described by a more advanced theory than quantum mechanics the security proof might not hold. This has, however, no consequences for implementations. The other premises can be placed in two categories. The first, containing only (1), leads to requirements for the devices which need to be addressed by the manufacturer. The second category (2), (3), (4-b), and (4-c) leads to requirements on the operational environment of the devices, which need to be addressed by the users, Alice and Bob.

(1) seems obvious and is most often not stated explicitly as an premise but only mentioned indirectly when describing the protocol. Moreover, it is important to note that DIQKD is possible with any number of devices that is above two. But there needs to be at least one device for each party. One large device that connects both labs contradicts the assumptions of a Bell test and renders compiling to premises (2) and (3) impossible. Further, the devices must give an unambiguous output when provided with an input and should be easy to identify even for non-experts. Defining a microscopic quantum object, e.g., an atom as an device is in principle possible for DIQKD in contrast to other device-independent applications (random number generation [9] or self-testing [10]). However, such a definition is not very useful since a quantum object will always be embedded in a bigger device holding and controlling it and hence this can be defined as the device without bothering about the actual quantum system. The well defined rounds are

necessary as the Bell test demands that for each input one always receives an output, otherwise the detection loophole will be open and invalidate the DI trust. Therefore, (1) directly transforms for a requirement for a system designed for DIQKD.

Now to the requirements that need to be addressed by the users, Alice and Bob. Restricting the communication of the devices for DIQKD is necessary, as it (2) ensures local measurements for the Bell test and (3) prohibits the possible malicious devices from simply leaking information to an eavesdropper. In many works, including [11], these two premises are combined to the demand for perfectly shielded rooms for Alice and Bob. However, such an ideal room is in practice impossible to realize without at least assuming some limitations on the devices. The biggest obstacle is that the two devices need to establish entanglement between them. This can be realized in different ways, but in all of them there is some physical connection to the outside-world. Prohibiting information leakage over this connection cannot be guaranteed without additional assumptions. Nevertheless, it is possible to build very secure rooms to limit the possibility of information leakage dramatically, dependent on the demands of the user.

Furthermore, trusted inputs (4-b) are necessary for QKD as well as for a Bell test. They are best provided by trusted random number generators, which are indeed a common demand for cryptographic application. However, here is not the explicit need of a true or quantum random number generator, one can also use any bit sequence which is unknown to the devices and potential eavesdroppers.

Finally, the last premise (4-c) summarizes all necessities for the extraction of a secret key from the recorded data. These are not always explicitly stated but are then implicitly still made. The authenticated classical public channel is needed to ensure that the DIQKD connection is between Alice and Bob and not relayed to an eavesdropper for, e.g., man-in-the-middle attacks. The trusted storage is needed to ensure the integrity of the recorded data. Storing in- and outputs only in the devices is not possible as they might be malicious and simply exchange the recorded inputs and outputs with a prerecorded data set. Although not discussed in detail here, the appropriate methods for error correction and privacy amplifications have to be used.

c. Proof-of-concept implementation The first step from the proposal to a real world application is a proof-of-concept implementation. Here, the goal is to show that the protocol can be implemented with the currently available technology. For such an experimental implementation some of the requirements can be relaxed, as the goal is not to send a secret message, but to show that this is in principle possible. This is especially true for the requirements that need to be addressed by the users. Thus, the main goal is to build two devices that fulfill requirement (1) and permit users to fulfill requirements (2), (3), (4-b), and (4-c).

As described in the main text, the presented QNL formed by the two atom traps enables exactly this. It consist of two independent devices. The devices are able to receive four, respectively two, different input values and respond with an unambiguous output. The heralded entanglement generation and event-ready measurement scheme allow for well defined rounds and closes the detection loophole in a Bell test. Thus it is compatible with all demands in (1). To further prove it is compatible with the other requirements they are fulfilled in a reasonable fashion, see the main text. This shows the suitability of the proof-of-concept implementation without the need of further argumentation, e.g., based on the physical model of the devices.

Appendix B: Atom-Photon Entanglement Generation

Both devices, i.e. atom traps, are characterized individually by analyzing the atom-photon entanglement generation process. The process starts by preparing the atom in the $5^2S_{1/2}|F=1, m_F=0\rangle$ state, denoted as $|1,0\rangle$, via optical pumping. Next, the atom is excited with a laser pulse that is resonant to the transition $5^2S_{1/2}|F=1\rangle \rightarrow 5^2P_{3/2}|F'=0\rangle$ and polarized parallel to the quantization axis (π-polarization). The temporal shape of the pulse is approximately Gaussian (22 ns FWHM). In the subsequent decay, the polarization of the photon that is emitted along the quantization (z-)axis becomes entangled with the atomic spin state, resulting in the following maximally entangled atom-photon state

$$\begin{aligned}|\Psi\rangle_{AP} &= 1/\sqrt{2}(|\downarrow\rangle_z|L\rangle + |\uparrow\rangle_z|R\rangle) \\ &= 1/\sqrt{2}(|\downarrow\rangle_x|H\rangle + |\uparrow\rangle_x|V\rangle),\end{aligned}$$

where $|\downarrow\rangle_z$ and $|\uparrow\rangle_z$ denote atomic spin states $|1,-1\rangle$ and $|1,+1\rangle$, $|L\rangle$ and $|R\rangle$ denote left- and right-circular photonic polarization states, and $|V\rangle$ and $|H\rangle$ denote vertical and horizontal linear photonic polarization states, respectively.

The success probability of the entanglement generation process, i.e. detection of a photon after an excitation pulse, equals 5.98×10^{-3} and 1.44×10^{-3} for Alice's device and Bob's device, respectively. Note that the lower photon detection probability for Bob's device is due to attenuation loss of approximately 50% in the 700 m optical fiber and

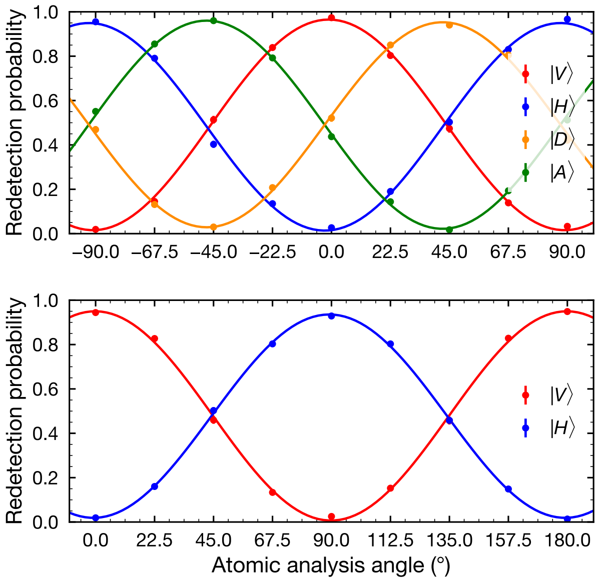

FIG. B.1. **Observation of atom-photon entanglement for Alice's device (top) and Bob's device (bottom).** The atomic analysis angle, i.e. readout polarization angle whereby 0° equals vertical, see equation (3) main text, is varied while measuring the photonic polarization in the H/V and D/A (only for Device 1) basis. Based on the fits the estimated fidelity of the entangled atom-photon state equals 0.952(7) and 0.941(7), for Alice and Bob, respectively.

the loss due to additional optical elements, including the beam splitter (90:10) for the local fluorescence detection and spectral filter shielding the read-out light, by another 50%, see Figure 2 of the main text.

The atomic spin state is analyzed after a delay of 25.6 µs and 16.7 µs, for Trap 1 and 2, respectively. This time allows for event-ready entanglement generation (two-way communication time between the labs equals approximately 7 µs) and provides rephasing of both the Larmor precession due to the magnetic bias field 57 mG and 168 mG along the y-axis and the transverse trap frequencies.

The atomic qubit is analyzed via a state-selective ionization scheme [12, 13], see main text Fig. 3b. There, a particular state of the atomic qubit is transferred to the $5^2P_{1/2}|F'=1\rangle$ depending on the polarization $\zeta = \cos(\gamma)V + e^{-i\phi}\sin(\gamma)H$ ($\gamma = \alpha$ for Alice's and $\gamma = \beta$ for Bob's device) by a 140 ns laser pulse from where it is ionized by a bright 473 nm laser pulse and thus leaves the trap. If the atom is still in the trap it is thus projected onto the state

$$|\text{Dark}\rangle = \sin(\gamma)|\downarrow\rangle_x - e^{-i\phi}\cos(\gamma)|\uparrow\rangle_x. \tag{B1}$$

In the experiment the presence of the atom is tested using fluorescence collection finally yielding the measurement outcome.

The atom-photon entanglement is analyzed by measuring the photonic polarization in the H/V (horizontal/vertical) and D/A (diagonal/anti-diagonal) basis, while varying the atomic analysis angle, i.e. readout polarization, as shown in Fig. B.1. In test runs, we observed 35259 and 20001 events for Alice's and Bob's side, respectively. The visibilities (Vis) of the measured states are obtained by fitting the data with sinusoidal functions. These result for Alice in visibilities of 0.942(14), 0.930(17), 0.942(13), and 0.954(19), for vertical $|V\rangle$, horizontal $|H\rangle$, diagonal $|D\rangle$, and anti-diagonal $|A\rangle$ photonic linear polarization states, respectively. For Bob, the fits give visibilities of 0.943(16) and 0.917(8), for $|V\rangle$ and $|H\rangle$ photonic linear polarization states, respectively.

To estimate a fidelity of the entangled state, one needs to take into account that a third atomic spin state can be populated $5^2S_{1/2}|F=1, m_F=0\rangle$ due to magnetic fields. Hence, assuming depolarizing noise in the 2x3 state space, a lower bound on the fidelity relative to a maximally entangled state is given by

$$\mathcal{F} \geq 1/6 + 5/6\overline{\text{Vis}}, \tag{B2}$$

with the average visibility $\overline{\text{Vis}}$, which results in estimated fidelities of 0.952(7) and 0.941(7), for Alice's device and Bob's device, respectively.

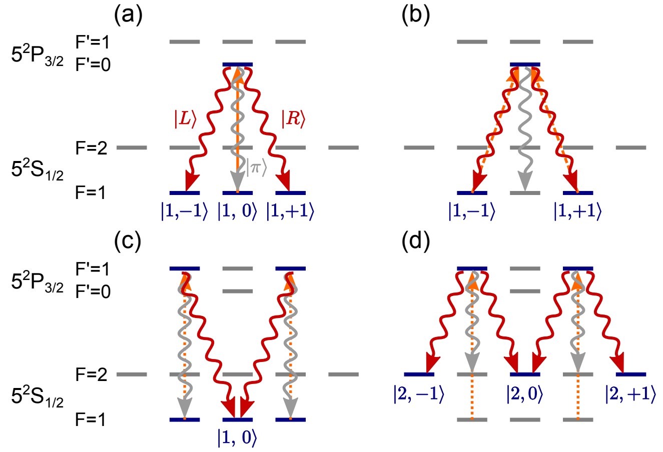

FIG. C.1. **Different branches of the excitation process in the level structure of** ^{87}**Rb.** (a) Intended generation of atom-photon entanglement in the spontaneous decay of the excited $5^2P_{3/2}|F'=0, m_{F'}=0\rangle$ level. The orange arrow indicates the prior excitation pulse. Photons polarized linearly along the quantization axis (π-decays, gray) are not detected by the single photon detectors. (b) In the case of an imperfect state preparation or a first decay, an excitation is possible due to polarization misalignment, or (c) off-resonant excitation. (d) When the $5^2\mathrm{P}_{3/2}|F'=1\rangle$ is excited, decays to $5^2\mathrm{S}_{1/2}|F=2\rangle$ level is possible.

Appendix C: Improving the Atom-Atom Entanglement Quality

The quality of the entangled atom-atom state depends on the generated atom-photon entanglement in both traps (see App. B) and on the performance of the Bell state measurement (BSM) on the photons. In order to understand these processes and subsequently improve on their performance, we modeled the excitation of a ^{87}Rb atom by a short laser pulse. Here, not only the physical properties of the system are considered, e.g., multilevel structure of the atom and the frequency broadening of the short laser pulse, but also imperfections of the experimental setup and procedure, such as imperfect polarization and state preparation.

In the intended atom-photon entanglement generation process (Fig. C.1(a)) the selection rules prohibit a second interaction with the π-polarized excitation laser. However, there are two effects that result in different types of emission. The first is caused by an experimental limitation: a small polarization misalignment of the excitation laser makes a second excitation possible, see Fig. C.1(b). Secondly, due to the small separation of the $5^2\mathrm{P}_{3/2}|F'=0\rangle$ to the $5^2\mathrm{P}_{3/2}|F'=1\rangle$ level off-resonant scattering via this level is possible (Fig. C.1(c),(d)). These effects lead to the emission of a second photon that perpetuates the atom-photon state and reduce its fidelity. Accordingly, this will be passed through by the swapping process also reducing the atom-atom state fidelity.

Beyond the effects reducing the fidelity of both the atom-photon and atom-atom states, there is a unapparent other effect reducing the fidelity of the two-photon interference based BSM. This process includes emission of a π-polarized photon followed by a regular excitation and decay. The π-polarized photon is not coupled into the single mode fiber and thus does not contribute to the atom-photon state, however, the temporal shape of the collected (second) photon is different than for a photon originating from a single excitation and emission process. This reduces the two-photon interference contrast, leading to a lower BSM fidelity and imperfect atom-atom state preparation.

A numerical simulation of the temporal behavior yields a time dependent photon emission (and thus detection) probability broken down for each of the different excitation processes described in Figure C.1. A complete and detailed description of the model used for the numerical simulation can be found in [14, 15]. Based on this result it is possible to optimize the two-photon acceptance time window for the BSM. The main finding is that the resulting entangled atom-atom state has the highest fidelity relative to the desired Bell state if only photons are accepted that are emitted after the end of the excitation pulse. This excludes the perpetuated atom-photon states as well as the effect of the imperfect state preparation, and increases the quality of the entanglement swapping operation.

While the first point follows directly from the simulated time dependent detection probability of the different excitation branches, the second is not that obvious. For this the following situation has to be considered: One atom emits only one photon, which is collected and detected, while the other one of the two atoms undergoes a two photon emission process first emitting a π-polarized photon and then being excited again emitting a second photon which is detected. If in this case one of the two detected photons is detected at an earlier time, especially during the excitation pulse, it can be assigned with very high probability to the atom emitting only one photon and the late photon to the atom with the two photon emission. Since the emission of the first π-polarized photon, in principle, allows the identification of the atom with the two photon process, the atom-atom state is not projected onto an entangled state

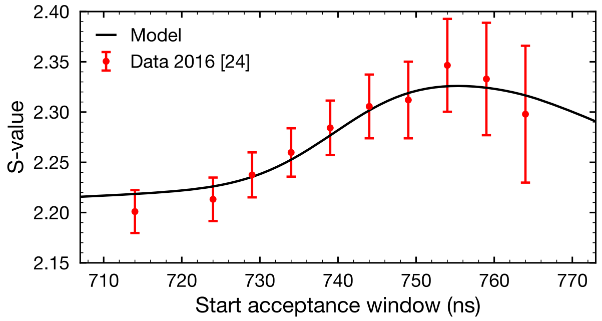

FIG. C.2. **CHSH S-value and relative event rate as a function of the starting time t_s of the acceptance time window.** The acceptance window ends with $t_e = 850$ ns. The numerical model is compared to experimental data collected for [16].

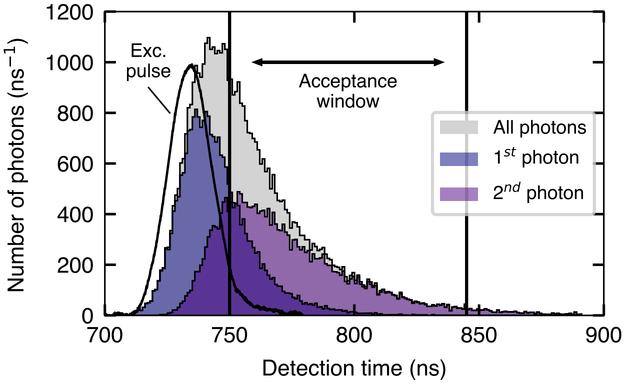

FIG. C.3. **Photon detection time histogram and acceptance window** In total 27% of the two-photon events are accepted, note that both photons should arrive within the acceptance window. The time on the x-axis in the plot is in relation to an trigger signal provided by the control electronics of the the experiment. The position of the excitation pulse compared to the detected photons represents the timing during the emission and not the detection process.

by the BSM.

Based on the outcome of the model (Fig. C.2), we define a two-photon acceptance time window of 95 ns that starts after the excitation pulse, as illustrated in Fig. C.3. While it drastically increases the entanglement fidelity, as shown in the simulation and the data presented in the main text, the shorter acceptance time window reduces the event rate by a factor of 4. Note that defining a smaller acceptance time window before the experiment does not lead to a ready-signal in the first place and thus does not open any kind of loophole, e.g., the detection loophole, in an Bell test.

For a complete analysis of the experiment, we also recorded the events outside of the time window, however, these events are not used in the DIQKD demonstration. The analysis of the complete dataset shows an increase of the interference contrast and atom-atom state fidelity for shorter time windows (Fig. C.4). The effect of excluding events with errors in the atom-photon entanglement generation is also observed in the read-out outcomes for both traps individually, as illustrated in Fig. C.5. For an ideally entangled atom-photon state, the ionization probability is 0.5, however, the processes reducing the atom-photon state fidelity, e.g., a second off-resonant excitation, lead to atomic states with higher ionization probabilities.

An even smaller time window might increase the atom-atom entanglement generation even further, thus leading to higher S and lower QBER which in turn result in an higher asymptotic key rate (Fig. C.6). However, this further reduces the event rate and increases the time needed for a measurement yielding a sufficient amount of events. More interesting for future experiments is the possibility of optimizing the excitation pulse shape in combination with narrow band filtering of the single photon frequency. A shorter excitation pulse, in combination with spectral filtering of off-resonant excitations, might lead to a more precise filtering of unwanted photons and an higher event rate.

To reach event rates high enough to generate a secure key using DIQKD for a finite block length [5], one has not only to consider the quality of the entanglement but also the generation rate. Thus, for finding the optimal acceptance

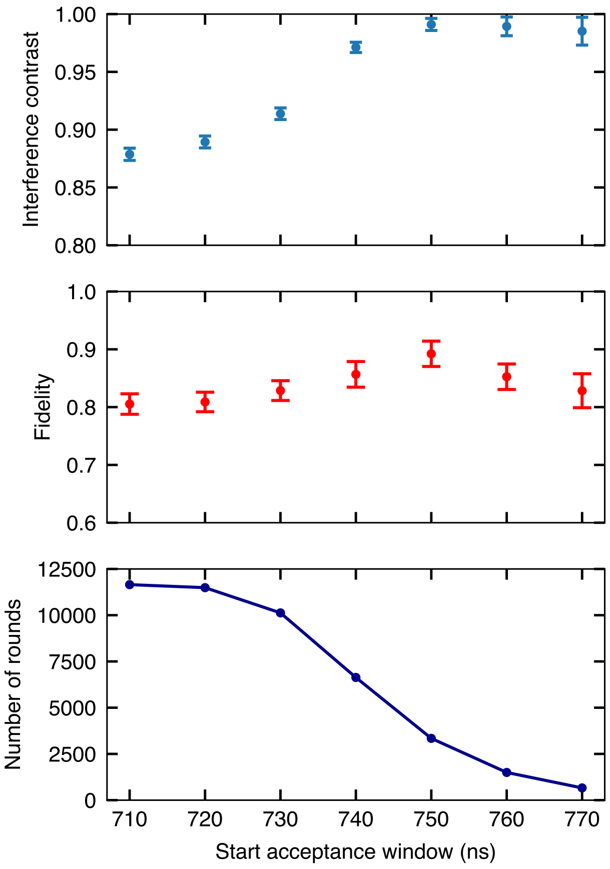

FIG. C.4. **Analysis of the data recorded during the DIQKD experiment.** Two-photon interference contrast, atom-atom state fidelity, and number of events depending on the starting time of the acceptance time window.

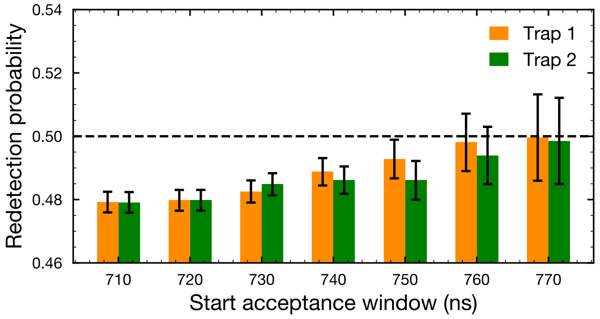

FIG. C.5. **Atomic state readout result for Trap 1 and 2 for varying start times of the acceptance window.** Perfect entanglement generation and readout would result in a redetection probability of 0.5. The data presented in the main text.

time window for such an experiment must consider the trade-off between them.

Appendix D: Estimating the expected secret key rate

A rigorous security analysis of practical DIQKD would require a finite-key analysis that takes into account the resources consumed and block-length considerations [17]. However, as mentioned in the main text, our experiment, which prioritises the establishment of swapped entangled trapped atoms 400 metres apart, has an intrinsic limitation on the event rate based on state-of-the-art technology. Consequently, there is a trade-off between the event-rate and

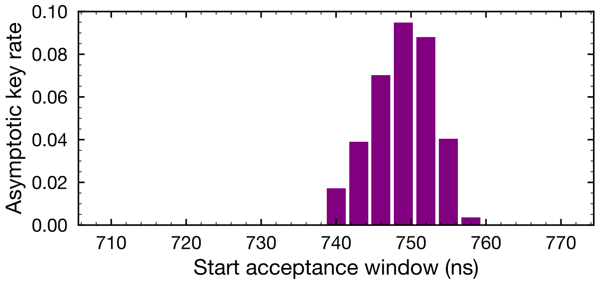

FIG. C.6. **Expected secret key rate for the robust DIQKD protocol for different t_s.**

TABLE I. **Summary of in- and output measurement correlations.** Number of rounds for each of the eight input setting combinations together with the number of rounds the devices gave correlated outcomes.

number of rounds $N_{X,Y}$				
	X=0	X=1	X=2	X=3
Y=0	448	425	389	434
Y=1	408	412	403	423
with correlated outputs $N_{X,Y}^{A=B}$				
	X = 0	X = 1	X = 2	X = 3
Y = 0	35	205	78	73
Y = 1	198	32	326	64
with anti-correlated outputs $N_{X,Y}^{A\neq B}$				
	X = 0	X = 1	X = 2	X = 3
Y = 0	413	220	311	361
Y = 1	210	380	77	359

separation of the laboratories, hence it is not realistic to demonstrate finite-key security based on known calculation method [17].

To that end, we estimate the expected secret key rate of the DIQKD experiment using standard Bayesian analysis; while we acknowledge that this is not the usual approach for QKD, it nevertheless gives a reliable estimate based on available data. Starting from the data summary listed in Tab. I, we model the random behaviour of S (its winning probability), Q_0, and Q_1 using Beta random variables, β_{win}, β_{Q_0}, and β_{Q_1}, respectively, which is in line with the self-testing statistical analysis reported in Ref. [10]. In particular, using a uniform prior, the (updated) posterior distributions are

$$\begin{aligned} \beta_{\text{win}} &= \text{Beta}(1357+1, 1649-1357+1), \\ \beta_{Q_0} &= \text{Beta}(35+1, 448-35+1), \\ \beta_{Q_1} &= \text{Beta}(32+1, 412-32+1), \end{aligned} \tag{D1}$$

where Beta(a, b) is the standard Beta distribution, and the winning probability is related to the CHSH value by $P_{\text{win}} = (S+4)/8$.

Then, to calculate the worst-case estimate of the expected secret key rate, we fix the tail errors of the updated Beta distributions to 3%; this means a 97% chance that each of the parameters would be higher (or lower) than a certain critical threshold. More specifically, we find $S \geq 2.4256$ and $Q_0 = Q_1 \leq 0.107$. Finally, using uniform settings (as was done in our experiment), we find that these critical values provide positive key rates.

Appendix E: Comparison to Other Experiments

Here we study the performance of the presented DIQKD setup (1) in relation to other experiments. To the best of our knowledge, two other experiments could fulfill the requirements of DIQKD with significant distance between

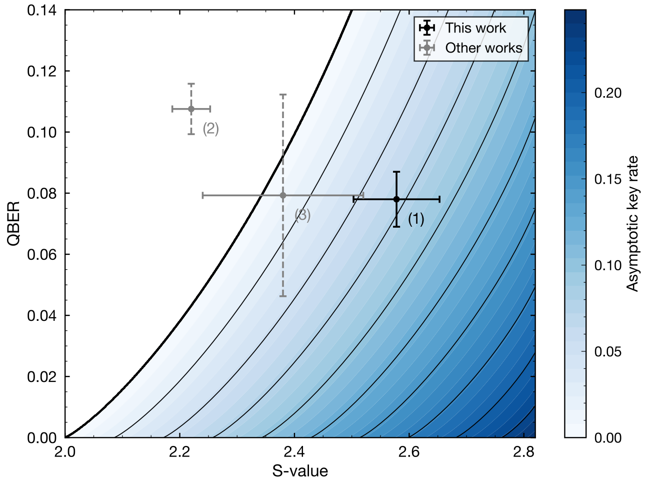

FIG. E.1. **Asymptotic key rate for the DIQKD system.** Expected secret key rate for varying CHSH S-value and QBER for the random key basis DIKQD protocol [11]. The asymptotic key rate for a perfect Bell violation and a zero QBER the implemented protocol with uniformly distributed measurement settings equals 0.25. The presented work (1) shows an expected secret key rate of 0.07 bits and lies well inside the positive region. For comparison, the results of Ref. [16] (2) and Ref. [18, 19] (3), which, to the best of our knowledge, are the only experiments that fulfill the requirements for DIQKD with significant distance between the two users. Note that (2) does not reach the positive key regime and the situation is indecisive for (3). To emphasise that the QBER was not measured for (2) and (3) the errors are plotted with dashed lines. The error bars indicate statistical errors of one standard deviation.

the two users: Ref. [16] (2) and Ref. [18, 19] (3). Note that these experiments are loophole-free Bell test and not aimed at QKD, therefore, the QBER was not observed. To still allow for some comparison, we estimate the QBER of these setups by via $Q = (1 - S/(2\sqrt{2}))/2$, assuming a depolarizing channel. With this method, the statistical errors of Q and S in (2) and (3) are perfectly correlated and hence should be interpreted differently from (1), for which the statistical errors are uncorrelated.

Fig. E.1 shows the asymptotic key rate for the random key basis protocol with respect to the S-value and QBER. The three setups are plotted at the observed (and estimated) positions. We conclude that (1) lies well within the positive key region (see Appendix D), while (2) is not able to generate positive asymptotic key rates. The situation is indecisive for (3).

Appendix F: Experimental Time Sequence

Fig. F.1 shows a detailed experimental time sequence after a successful entanglement generation try. The sequence starts by sending a "ready" signal to the devices. In both devices, an electronic time delay allows for a complete oscillation period of the trap frequency and Larmor precession (see Appendix B). After rephasing, two and one random bits are generated at Alice's and Bob's side, respectively, to select the measurement setting. Subsequently, the atomic states are analysed by a state-selective ionization scheme. The binary result of this scheme is verified by atomic fluorescence collection. On Bob's side, to prevent leakage of the device output via the quantum channel, a 5 ms delay is implemented before the fluorescence readout to close the quantum channel with a free-space shutter (see Fig. 2 of the main text) and the trap is always emptied before reopening the shutter.

The measurement events are not space-like separated during this experiment; the security of a DIQKD protocol is not increased by closing the locality loophole [3, 20]. The security is already guaranteed under assumptions (2) and (3), which restrict information leakages from the devices. More specifically, it is enforced that no information leaks out of a closed environment around the users—in our case the two laboratories—and hence the measurement outcomes of both devices cannot influence each other. This relaxes the requirements on space-like separation for the security of DIQKD, i.e., not closing the locality loophole does not open up possibilities for Eve to gain more information about the key.

In Ref. [16] the locality loophole was closed with the same setup as used for the presented experiment. Therefore, the absolute timings of the measurements in Device 1 and 2 were matched by tuning the optical dipole trap depths and magnetic field strengths. Furthermore, to complete the fast ionization based state readout ($< 1\mu$s), channel electron multipliers were used for directly detect the ion and electron fragments; making the slow ($\sim$ 60 ms) fluorescence

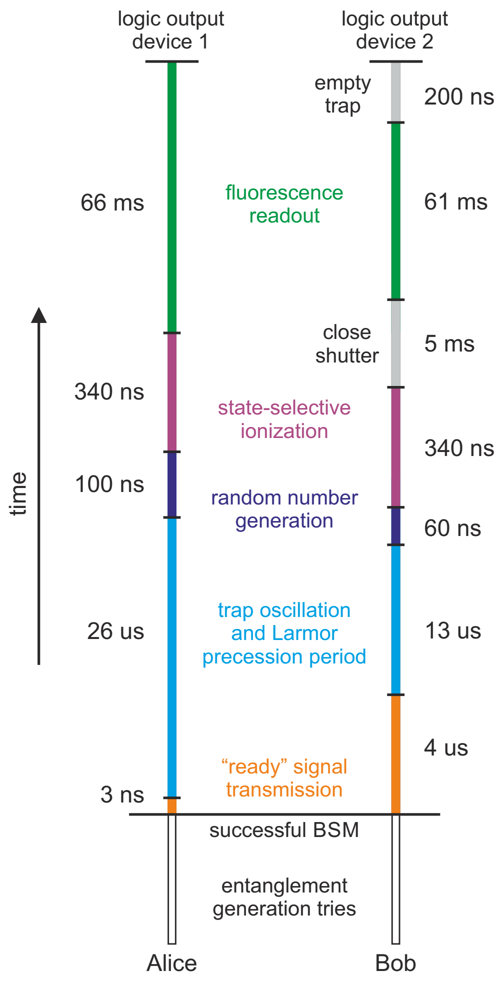

FIG. F.1. **Detailed time sequence after a successful BSM.**

collection unnecessary.

Appendix G: Stability of the Setup

The stability of the DIQKD system is crucial. Due to the relatively low event rate, it is required to run the protocol for several days to make confident statements about the system's performance. Therefore, many parameters in both atom trap setups are actively stabilized or automatically optimized. Furthermore, maintenance shifts are needed to optimize, among others, laser frequency locks, laser pulse powers, magnetic fields, and polarization drifts in fibres. The measurement run presented in the main text was paused six times for scheduled maintenance.

Here we assess the stability of the system by observing three parameters: the event rate, polarization drifts in the 700 m long fibre, and the individual device outputs, as illustrated in Fig. G.1. First, the event rate of the system over the complete measurement run is analysed by observing the number of completed rounds over time; the (almost) linear behaviour indicates a stable event rate. The event rate can fluctuate slightly by changes in ratio of time that an atom is present in both single-atom traps and drifts in efficiency of the entanglement generation process; both influenced by laser pulse power fluctuations. Next, we analyse the polarization changes in the 700 m long fibre, which is automatically compensated for every 3–5 minutes. For this, we measure, before every optimization run, the Stokes vector of a classical laser beam at the output of the long fibre, where the input to the long fibre was V or $+45$ polarized. We observe polarization drifts in the 700 m long fibre of $< 0.5\%$. The optimization is based on an gradient decent algorithm whereby the polarization is controlled with an fibre polarization controller [21]. Finally, the individual outputs of the devices are analysed over time. For this, we bin the device outputs per 250 rounds and

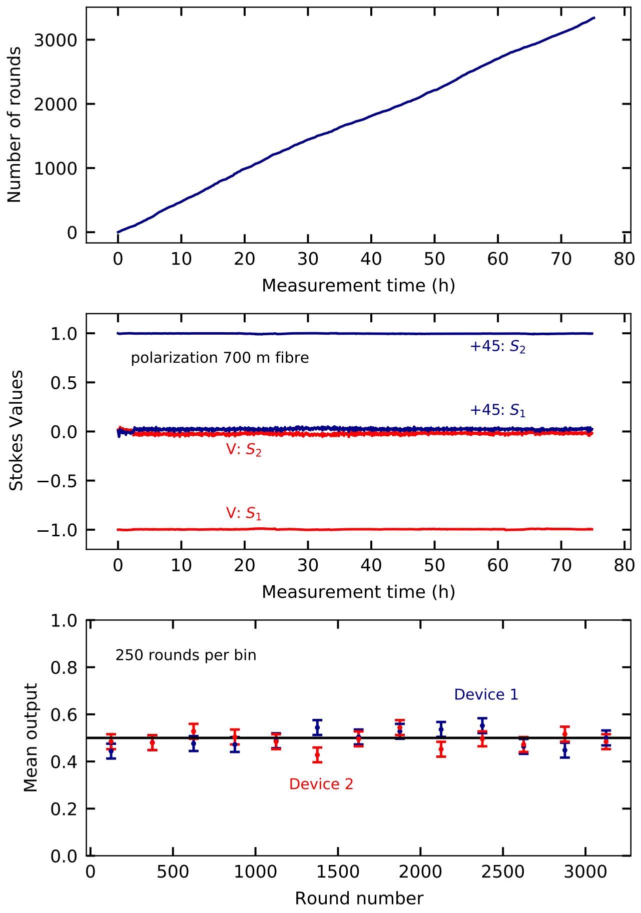

FIG. G.1. **Stability of the DIQKD system.** From top to bottom: number of measurement rounds over the measurement time, polarization drifts in the 700 m long fibre channel over the measurement time, and the mean output of the devices per 250 rounds where the device output $\uparrow$ ($\downarrow$) corresponds to the value 0(1).

calculate the mean output where the device output $\uparrow$ ($\downarrow$) corresponds to the value 1(0). Following this method, ideal devices have a mean output of 0.5. For our system, a reduced atom-photon entanglement fidelity results in a lower mean output, of which we do not see any sign.

Appendix H: Towards DIQKD Applications

For a practical demonstration of DIQKD, the employed apparatus should:

1. show entanglement quality enabling for a positive key rate;

2. obtain entanglement over distances relevant for cryptography; and

3. reach entanglement rates allowing for key distribution on practical timescales.

Currently, generating an high-quality atom-atom entanglement event per approximately 80 s over a distance of 400 m, the setup does not yet fulfil the third requirement. Hence, next steps involve improving the entanglement generation rate.

Three realistic improvements on the current setup can increase the event rate by an order of magnitude. First, preparing more than one entangled state in the swapping process, here the $|\Psi^-\rangle$ state. This is already possible with the current setup, but its quality needs to be improved. Second, using superconducting nanowire single-photon detectors with quantum detection efficiencies of $> 90\%$, which could quadruple the entanglement generation rate.

Finally, improving the atom-photon entanglement generation quality and hence reducing the requirement of temporal filtering in the BSM, see Appendix C.

Beyond these incremental improvements, neutral optically trapped atoms are an ideal candidate to scale up the number of individually controllable atom traps and hence enable for temporal multiplexing of the entanglement generation process. By employing micrometer spaced trapping potentials, it is possible to realize defect free arrays of single atoms while allowing for individual control of the trapping sites.

Various approaches exist to realize multi-dimensional trap arrays, for example, using a spatial light modulators [22], acousto-optical deflectors [23], or microlens array [24]. Currently, state-of-the-art trapping techniques allow for individual storage and control of > 100 single atoms, potentially increasing the event rate by orders of magnitude.

Another advantage of employing arrays of single atom traps is the possibility to implement entanglement distillation protocols when sharing various entangled atom-atom pairs between two setups [25]. This provides an promising platform to realize a quantum repeater.

---

[1] A. Acín et al., "From Bell's Theorem to Secure Quantum Key Distribution," Phys. Rev. Lett. **97**, 120405 (2006).

[2] A. Acín et al., "Device-Independent Security of Quantum Cryptography against Collective Attacks," Phys. Rev. Lett. **98**, 230501 (2007).

[3] S. Pironio, "Device-independent quantum key distribution secure against collective attacks," New J. Phys. **11**, 045021 (2009).

[4] U. Vazirani and T. Vidick, "Fully Device-Independent Quantum Key Distribution," Phys. Rev. Lett. **113**, 140501 (2014).

[5] R. Arnon-Friedman et al., "Practical device-independent quantum cryptography via entropy accumulation," Nat. Commun. **9**, 459 (2018).

[6] J.-Å. Larsson, "Loopholes in Bell inequality tests of local realism," Journal of Physics A: Mathematical and Theoretical **47**, 424003 (2014).

[7] Valerio Scarani, Bell Nonlocality (Oxford University Press, 2019).

[8] Rotem Arnon-Friedman, Renato Renner, and Thomas Vidick, "Simple and tight device-independent security proofs," SIAM Journal on Computing **48**, 181–225 (2019).

[9] S. Pironio et al., "Random numbers certified by Bell's theorem," Nature **464**, 1021 (2010).

[10] Jean-Daniel Bancal, Kai Redeker, Pavel Sekatski, Wenjamin Rosenfeld, and Nicolas Sangouard, "Self-testing with finite statistics enabling the certification of a quantum network link," Quantum **5**, 401 (2021).

[11] René Schwonnek, Koon Tong Goh, Ignatius W. Primaatmaja, Ernest Y. Z. Tan, Ramona Wolf, Valerio Scarani, and Charles C. W. Lim, "Device-independent quantum key distribution with random key basis," Nature Communications **12**, 2880 (2021).

[12] T. van Leent et al., "Long-Distance Distribution of Atom-Photon Entanglement at Telecom Wavelength," Phys. Rev. Lett. **124**, 010510 (2020).

[13] N. Ortegel, "State readout of single rubidium-87 atoms for a loophole-free test of bell's inequality," PhD thesis, Ludwig-Maximilians-Universität München (2016).

[14] Julian Hofmann, "Heralded Atom-Atom Entanglement," PhD Thesis, Ludwig-Maximilians-Universität München (2014).

[15] Kai Redeker, "Entanglement of single rubidium atoms: from a bell test towards applications," PhD Thesis, Ludwig-Maximilians-Universität München (2020).

[16] W Rosenfeld et al., "Event-Ready Bell Test Using Entangled Atoms Simultaneously Closing Detection and Locality Loopholes," Phys. Rev. Lett. **119**, 010402 (2017).

[17] Ernest Y. Z. Tan, Pavel Sekatski, Jean-Daniel Bancal, René Schwonnek, Renato Renner, Nicolas Sangouard, and Charles C. W. Lim, "Improved diqkd protocols with finite-size analysis," (2020), arXiv:2012.08714 [quant-ph].

[18] B. Hensen et al., "Loophole-free Bell inequality violation using electron spins separated by 1.3 kilometres," Nature **526**, 682 (2015).

[19] B. Hensen et al., "Loophole-free Bell test using electron spins in diamond: second experiment and additional analysis," Scientific Reports **6**, 30289 (2016).

[20] Nicolas Brunner, Daniel Cavalcanti, Stefano Pironio, Valerio Scarani, and Stephanie Wehner, "Bell nonlocality," Rev. Mod. Phys. **86**, 419–478 (2014).

[21] W. Rosenfeld et al., "Towards Long-Distance Atom-Photon Entanglement," Phys. Rev. Lett. **101**, 260403 (2008).

[22] Daniel Barredo, Sylvain De Léséleuc, Vincent Lienhard, Thierry Lahaye, and Antoine Browaeys, "An atom-by-atom assembler of defect-free arbitrary two-dimensional atomic arrays," Science **354**, 1021–1023 (2016).

[23] Manuel Endres, Hannes Bernien, Alexander Keesling, Harry Levine, Eric R Anschuetz, Alexandre Krajenbrink, Crystal Senko, Vladan Vuletic, Markus Greiner, and Mikhail D Lukin, "Atom-by-atom assembly of defect-free one-dimensional cold atom arrays," Science **354**, 1024–1027 (2016).

[24] Daniel Ohl de Mello, Dominik Schäffner, Jan Werkmann, Tilman Preuschoff, Lars Kohfahl, Malte Schlosser, and Gerhard Birkl, "Defect-free assembly of 2d clusters of more than 100 single-atom quantum systems," Phys. Rev. Lett. **122**, 203601 (2019).

[25] Norbert Kalb, Andreas A Reiserer, Peter C Humphreys, Jacob JW Bakermans, Sten J Kamerling, Naomi H Nickerson,

Simon C Benjamin, Daniel J Twitchen, Matthew Markham, and Ronald Hanson, "Entanglement distillation between solid-state quantum network nodes," Science **356**, 928–932 (2017).
